# Annotations

**Published:** 1898-06-18

**Authors:** 


### Annotations.

### Lead in the Potteries.

In an eloquent and fervid letter to the *Times*, the Hon. Sydney Holland urges the iniquities of the pottery trade and the horrors of lead poisoning. It must be admitted that most of the severe cases of plumbism have had some warning, and that such cases would be but rare if, on the first warning, people would give up working amongst lead. When competition for a livelihood is so keen, it seems hard to say that it is a workman's own fault if he continues at work after his first attack; but the reports of the factory inspectors show that "the higher wages for lead workers induces them to run all risks." This has to be borne in mind in considering the remedy suggested in the letter referred to, namely, putting lead poisoning among the "accidents" for which an employer will be responsible under the Workmen's Compensation Act, for it is pretty certain that the first effect of such a plan would be to cause the discharge of all who have shown themselves susceptible to lead. This would undoubtedly be looked on by the workers as a hardship. We have, indeed, very little doubt that lead poisoning could be reduced to very small dimensions if both employers and employed would strictly and loyally carry out the rules laid down for the governance of the various dangerous trades. The "susceptibles" must, however, be weeded out, and there lies the hardship.

### The Diagnosis of Phosphorus Poisoning.

A question recently asked in the House of Commons by Mr. John Burns touches a large and very difficult problem. What is to be done to the doctor who does not know? In other words, what is to be done to the man who avoids the fulfilment of the statutory duty of notifying a disease by the plea that he did not recognise its nature? The question put had to do with the recently reported case of phosphorus poisoning in the match trade. It had been stated by the Home Secretary that a medical man had seriously failed in his duty in this case, and Mr. Burns asked whether the Home Secretary would bring his conduct before the Royal College of Surgeons. But what is the College of Surgeons to do? The same sort of difficulty has occurred again and again in regard to the notification of fevers. The Act says that notification is to be made when the doctor is "aware" that the patient is suffering from the disease in question. But when is he aware? And what is to be done with a medical man who blandly asks how he could possibly be "aware" that a child was suffering from scarlet fever until all the symptoms had developed, one of the symptoms being desquamation, or who maintains that he cannot be aware of the existence of typhoid fever till spots appear, or of small-pox until an umbilicated pustule declares indubitably the nature of the case? It is clear enough that in the vast majority of cases such a delay in arriving at a diagnosis would betray gross incompetence; yet it cannot be denied that cases do occur now and again in which it is only by waiting for the full evolution of events that certainty can be arrived at. Generally, in a case of phosphorus necrosis, it is easy enough to come to a conclusion as to the origin of the disease, but in regard to some cases, especially if separated from their history and surrounding, it would puzzle the whole College to decide positively between phosphorus, clumsy dentistry, and the necrosis which sometimes follows typhoid fever. We doubt, then, whether much would be got by referring the matter to the College of Surgeons. Each case must stand by itself, and it must be the evidence of those who have themselves seen it that must decide whether or not the law has been broken. Until the law is shown to have been broken, the College is not likely to intervene, and in no case is it likely to lay down any hard and fixed rule that a man who fails to diagnose phosphorus necrosis is, of necessity, to blame.

### Ice-Creams Again!

During the past week various articles in the public press have once again drawn attention to the evils arising from the filthy surroundings amid which the trade in ice cream is carried on. We are told of the enormous number of microbes which are to be found in some of the ices which are sold in the streets; of their disgusting nature, the microbes being, in fact, to a large extent derived from excremental material; of the dirty trick of blowing out the contents of the eggs employed in making the ices so as to save the shell, unbroken egg shells being saleable for use in shooting galleries; and worse than all, we are told of the places in which the ices are made and the places where the materials are stored, sometimes even under beds tenanted by sick people. Then we are asked to consider the sort of people who carry on this trade, the sort of "cleansing" to which the utensils and the glasses employed are subjected, and we are very properly urged to demand that a trade so full of possibilities of evil shall be placed by law under the strict control of the local authorities. But all this is old. In 1893 Dr. Campbell Munro, medical officer of health for Renfrewshire, reported a very considerable outbreak of typhoid fever, the diffusion of which was traced to the sale of ice-creams. These had been prepared on premises where there was an unreported case of the disease, the patient being a girl who during a considerable part of the time that she was ill had been in intimate contact with the business. Then in 1894, Dr. Harris, the well-known medical officer of health for Islington, caused samples of ice-creams sold in the streets in North London to be examined by Dr. Klein, and personally inspected the premises in which the stuff was manufactured. In that report we find exactly the same points raised that have again been brought up—the blowing of the eggs, the dirty process of manufacture, and the evil smelling sleeping rooms in which the product was stored, and finally the swarms of bacteria which were found in the ice-creams taken from the barrows in the streets. All was then just as it is now, or, to put the matter more forcibly, all remains now just as it was then, notwithstanding that then, four years ago, there were the same resolutions that we hear of now, that "representations should be made to the Local Government Board, urging them to introduce legislation to empower local authorities to register the vendors of these articles." We quote from a paper four years' old, and now, even as we write, we read in an evening paper, as the last pronouncement on the subject, "that a number of important suggestions have been made on the subject, the one almost generally accepted being action by the Local Government Board to introduce legislation giving the local authorities power to license vendors and regulate the conditions under which the cream is made and sold." This after four years! Exactly the same thing over again! Truly we are a patient race. Meanwhile, what about the children by whom these ices are consumed?

